# IL-4-Induced Gene 1: A Potential Player in Myocardial Infarction

**DOI:** 10.31083/j.rcm2509337

**Published:** 2024-09-20

**Authors:** Rui Shen, Yan Ding, Qian Dong, Yue Wang, Jian Yu, Chengliang Pan, Yifan Cai, Zhiyang Li, Jiangmei Zhang, Kunwu Yu, Qiutang Zeng

**Affiliations:** ^1^Department of Cardiology, Union Hospital, Tongji Medical College, Huazhong University of Science and Technology, 430022 Wuhan, Hubei, China; ^2^Hubei Key Laboratory of Biological Targeted Therapy, Union Hospital, Tongji Medical College, Huazhong University of Science and Technology, 430022 Wuhan, Hubei, China; ^3^Hubei Provincial Engineering Research Center of Immunological Diagnosis and Therapy for Cardiovascular Diseases, Union Hospital, Tongji Medical College, Huazhong University of Science and Technology, 430022 Wuhan, Hubei, China

**Keywords:** myocardial infarction, IL4I1, LAAO, macrophage, CD4+ T cell, ferroptosis, immunometabolism

## Abstract

Myocardial infarction (MI), a severe outcome of cardiovascular disease, poses a serious threat to human health. Uncontrolled inflammation and excessive cardiomyocyte death, following an infarction event, significantly contribute to both the mortality rate and complications associated with MI. The protein IL-4-induced gene 1 (IL4I1 or FIG1) serves as a natural inhibitor of innate and adaptive immunity, playing a crucial role in CD4+ T cell differentiation, macrophage polarization, and ferroptosis inhibition. Previous studies have linked IL4I1 to acute MI. This review summarizes evidence from both basic and clinical research, highlighting IL4I1 as a critical immunoregulatory enzyme that not only regulates inflammatory responses, but also potentially mitigates MI-induced damage.

## 1. Introduction

Despite recent advances in treatment, myocardial infarction (MI), a severe form of coronary heart disease, remains a leading cause of morbidity and mortality worldwide [1, 2, 3]. The inflammatory response following MI is crucial for cardiac repair and ventricular remodeling [4]. After infarction, necrotic cardiac cells release damage-associated molecular patterns that promote sterile inflammation [5], leading to the infiltration of multiple activated immune cells within the infarcted area to participate in tissue repair. However, prolonged and uncontrolled inflammatory responses can enlarge the infarction and contribute to adverse remodeling [4]. Thus, a proper transition from pro-inflammatory to anti-inflammatory responses post infarction is therefore crucial for cardiac healing and improved prognosis.

**Fig. 1.**
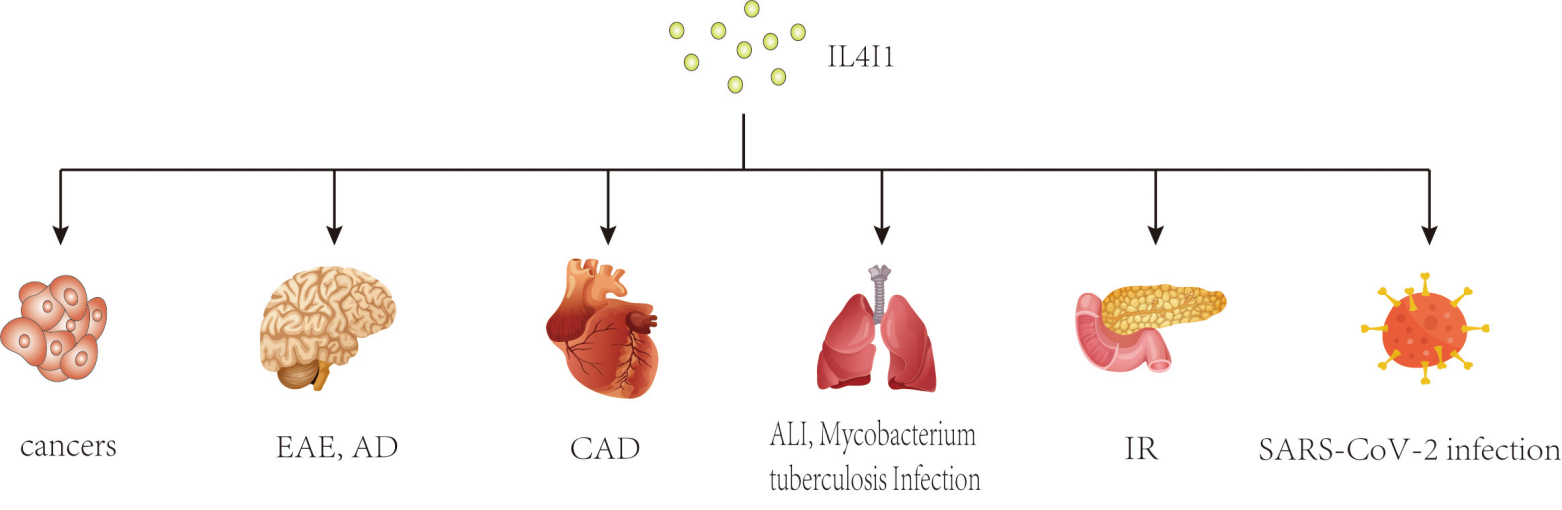
**Potential effects of IL4I1 on various diseases**. This diagram illustrates the broad impact of IL4I1, an immunosuppressive enzyme, across multiple disease states. As an immunosuppressive enzyme, IL4I1 has been studied extensively in cancers and inflammatory diseases. IL4I1, IL-4-induced gene 1; EAE, experimental autoimmune encephalomyelitis; AD, Alzheimer’s disease; CAD, coronary artery disease; ALI, acute lung injury; IR, insulin resistance.

As a metabolic immune checkpoint protein, interleukin-4-induced gene 1 (IL4I1 or FIG1) has been extensively studied in human cancers and inflammatory diseases. As a protein-coding gene, *IL4I1* is mainly expressed by antigen presenting cells including dendritic cells, macrophages, and B lymphocytes [6]. The protein encoded by this gene acts as an enzyme metabolizing amino acids, with a preference for common aromatic L-amino acids, and has immunomodulatory functions both *in vivo* and *in vitro* [7]. Studies have demonstrated that IL4I1 protein inhibits the effector T cell proliferation, promotes regulatory T cell (Treg) differentiation, and enhances M2 macrophage polarization depending on its enzymatic activity [8, 9, 10, 11]. Of note, a previous clinical study revealed that *IL4I1* mRNA expression levels were significantly reduced in peripheral blood mononuclear cells of patients with acute myocardial infarction [12]. Given these effects, it is reasonable to assume that IL4I1 may be involved in MI pathophysiology.

## 2. The Futures and Functions of IL4I1

First discovered in 1997 by Chu *et al*. [13], IL4I1 is a novel immunomodulatory enzyme that functions as an early IL-4-inducible gene in B cells. Subsequent research revealed that IL4I1 is also expressed in macrophages, dendritic cells, and T cells, suggesting IL4I1 is a versatile immunomodulator [6, 9, 14]. The human *IL4I1* gene is located on chromosome 19q13.3-13.4, a region implicated in susceptibility to systemic lupus erythematosus, rheumatoid arthritis, multiple sclerosis, and insulin-dependent diabetes mellitus, raising the possibility that IL4I1 may play a role in inflammatory diseases [13, 15, 16, 17]. The IL4I1 protein is highly expressed in immune organs and tissues such as the thymus, spleen and lymph nodes, while its expression is significantly lower in the heart and other nonimmune organs [13, 15]. Furthermore, IL4I1 proteins of humans and mice share a remarkable similarity, with the exception of the C-terminal region [13, 15]. This amino acid sequence includes a putative secretion signal peptide and a structure resembling flavoproteins which is essential for its catalytic activity [13, 15]. Beyond its secretion from cells, IL4I1 may also reside in lysosomes, displaying a unique preference for an acidic pH [18]. Enzymological characterization revealed that IL-4I1 has L-amino acid oxidase (LAAO) activity, preferentially oxidizing common aromatic L-amino acids such as phenylalanine, tyrosine, and tryptophan into corresponding ketoacids, while simultaneously producing hydrogen peroxide and ammonia [7, 18, 19].

In recent years, a growing body of research has focused on the role of IL4I1 in immune responses. Previous studies have shown that IL4I1 is highly expressed in various human cancers, including ovarian, colorectal, melanoma, head-neck cancers, and B-cell lymphoma, where it may facilitate immune escape and is associated with a poor prognosis [20, 21, 22, 23, 24, 25]. Interestingly, changes in IL4I1 expression have also been observed in inflammatory diseases such as pulmonary Aspergillus fumigatus infection, type 2 diabetes mellitus, autoimmune demyelinating diseases, and inflammatory bowel disease [26, 27, 28, 29] (Fig. 1). As an immunoregulator, IL4I1 inhibits the proliferation of effector T cells, promotes the differentiation of regulatory T cells from naïve CD4+ T cells, and enhances the polarization of M2 macrophages, thereby playing a key role in the progression of these diseases [8, 9, 10, 30]. Further research has identified several mechanisms of IL4I1’s immunomodulating activity, including essential amino acid consumption, catabolite generation, and direct interaction with undefined surface receptors [30, 31, 32]. Moreover, IL4I1 has antibacterial properties that extend beyond its initial immunoregulatory effects. Puiffe *et al*. [33]discovered that IL4I1 inhibits the growth of both gram-negative and gram-positive bacteria in *vitro* and *in vivo*. Taken together, these findings underscore the multifunctional nature of IL4I1 and highlight the need for further investigation.

## 3. IL4I1 and CD4+ T Cell Differentiation

Recent studies have identified T helper 17 (Th17) cells and regulatory T cells (Tregs) as two distinct subtypes of the T helper cell lineage that play important roles in immune system regulation through of diverse cytokine production [34, 35]. The Th17 cells express retinoic acid-related orphan receptor γt (ROR-γt) and secrete pro-inflammatory cytokines—including interleukin (IL)-17, tumor necrosis factor-α (TNF-α), and IL-6, contributing to several chronic inflammatory disorders [36]. In contrast, Treg cells, marked by the expression of the forkhead/winged helix transcription factor (Foxp3), maintain immune balance and stability by releasing IL-10 and transforming growth factor-β, which suppress the activity of other immune cells, including Th17 cells [37]. An imbalance between Th17 and Treg can significantly affect the onset, progression and prognosis of inflammatory and autoimmune diseases [38].

Researches have demonstrated that IL4I1, secreted by antigen-presenting cells, can regulate the proliferation and differentiation of CD4+ T cells via a mechanism involving down-regulated T-cell receptor (TCR) signaling [9, 30, 39]. These findings suggest that the LAAO may play an important regulatory role in the adaptive immune response [9, 30, 39]. As shown in a previous study, IL4I1 limits Th17 cell differentiation by reducing ROR-γt expression and induces its own production under Th17 polarization conditions [10]. Interestingly, Annunziato *et al*. [40] observed that IL4I1 expression was upregulated in Th17 cells, impairing their ability to proliferate and produce IL-2, a critical T cell growth factor. This increase in IL4I1 was associated with a decrease in CD3ζ chain expression and consequent disruption to the molecular pathway [14]. Moreover, the researchers also noted that IL4I1 maintains high levels of Tob1 expression in human Th17 cells [40]. As a member of the Tob/B-cell translocation gene antiproliferative protein family, Tob 1 prevents cell-cycle progression mediated by TCR stimulation [40].

In contrast, IL4I1 promotes the Treg differentiation from naïve CD4+ T cells [10]. A study by Cousin and his colleagues [10] provided supportive evidence through T-cell culture assays, revealing that IL4I1 induces the generation of both human and mouse Treg cells *in vitro*. In addition, Scarlata *et al*. [41] reported that IL4I1 is expressed in human Aiolos+, but not Helios+, FoxP3+ Tregs, suggesting that this differential expression may limit their conversion into Th17 cells. Notably, Aiolos+ FoxP3+ Tregs are considered as inducible Tregs and have the capability to give rise to Th17 cells due to changes in the microenvironment [42, 43, 44]. However, the transformation of Th17 and Treg cells is still a controversial issue.

Amino acid-depleting enzymes, including indoleamine 2,3 dioxygenase, tryptophan dehydrogenase, and inducible nitric oxide synthase, have been shown to impair T-cell activation and inhibit T-cell responses both *in vitro* and *in vivo* [45, 46, 47]. Essential amino acid consumption by IL4I1 in the local environment at least partially contributes to its effects on Th17 and Treg cells, which are associated with the mammalian target of rapamycin (mTOR) signaling pathway [48]. The ubiquitous mTOR is a serine/threonine kinase that regulates the metabolism, proliferation, and differentiation of T cells [49]. Specifically, mTOR complex 1 (mTORC1) promotes the expansion of Th1 and Th17 cells, while both mTORC1 and mTORC2 inhibit the development of Treg cells [49].

As a toxic metabolite, H_2_O_2_ can mediate the antiproliferative effect of IL4I1 [9]. Previous study has shown that H_2_O_2_ can induce apoptosis in human memory CD4+ and CD8+ T cells through the mitochondrial programmed cell death pathway [50]. Moreover, a recent study pointed that IL4I1, secreted from antigen presenting cells into the synaptic cleft, exerts its inhibitory effect on T cells by binding to an unidentified surface receptor [30]. The transmembrane Protease, Serine 13 (TMPRSS13) expressed by various immune cell types, was identified as a candidate receptor for IL4I1; their interaction may be important to IL4I1-mediated immunosuppression [51]. Taken together, these findings demonstrate that IL4I1 exerts different effects on Th17 and Treg cells through its enzymatic activity and receptor binding interactions, playing a crucial role in immune diseases by modulating the Th17/Treg balance.

## 4. IL4I1 and Macrophage Polization

Macrophages can be classified into two distinct subsets based on surface-marker expression, each with different functions: classically activated (M1) macrophages and alternatively activated (M2) macrophages [52, 53]. The M1 macrophages are induced by lipopolysaccharide, either alone or in combination with Th1 cytokines [52]. These can include interferon-γ or TNF-α, and primarily influence pro-inflammatory activities while playing a vital role in combating infections [52]. In contrast, M2 macrophages, which are polarized by Th2 cytokines including IL-4 and IL-13, exert anti-inflammatory effects, and participate in both tissue repair and regeneration [53]. Moreover, macrophages exhibit remarkable plasticity, allowing them to rapidly change their phenotype in response to the surrounding microenvironment and thereby regulate the balance between the pro-inflammatory and anti-inflammatory responses [54, 55].

The protein IL4I1 is mainly generated by dendritic cells and macrophages under inflammatory conditions [6]. Yue *et al*. [8] discovered that IL4I1expression in bone marrow-derived macrophages could be induced by Th1 and Th2 cytokines in two distinct patterns, with higher levels of IL4I1 protein in M2 macrophages when compared to M1 macrophages. Gene expression analysis revealed that IL4I1 contributed to the modulation of macrophage programming by influencing the phosphorylation of signal transducer and activator of transcription (STAT)3 and STAT6, which are key transcription factors involved in the polarization of macrophages into M1 and M2 classes [8, 56, 57]. Furthermore, overexpression of IL4I1 promotes the expression of M2 markers including found in inflammatory zone 1 (Fizz1), arginase 1 (Arg1), chitinase 3-like 3 (YM-1), and mannose receptor (MR) and inhibits the expression of M1-associated cytokines (TNF-α, IL-1β, and IL-12p40) [8]. Strikingly, tumor-associated macrophages, which invade tumor tissue and are generally considered M2 type, were found to express the immunosuppressive enzyme IL4I1 in numerous cancers [24, 58]. Moreover, a recent study by Gao *et al*. [59] observed that IL4I1 mediated the aggravation of lipopolysaccharide-induced acute lung injury by jumonji domain-containing protein 3 (JMJD3) via regulating the M1/M2 ratio. In an integrated investigation of mononuclear phagocytes across human healthy and diseased tissues, IL4I1+ macrophages established an immunosuppressive environment through the tryptophan degradation and promoted the accumulation of Tregs in tumors [60]. Altogether, it is safe to assume that IL4I1 is an important factor that influences the immune response by regulating macrophage polarization.

## 5. Role of IL4I1 in Ferroptosis

Ferroptosis, an iron-dependent form of regulated cell death, is characterized by the massive accumulation of lipid hydroperoxides [61, 62]. Similar to other types of cell death, ferroptosis may also influence the pathological processes behind MI [61, 62]. Modulation of ferroptosis involves several different cellular metabolic pathways including redox homeostasis, mitochondrial activity, and the metabolism of iron, amino acids and lipids [63].

When secreted, IL4I1 becomes active in the extracellular environment, and regulates neighboring cells by inducing changes in the local microenvironment [9]. As a major aryl hydrocarbon receptor (AHR)-activating enzyme, IL4I1 promotes tumor cell motility and suppresses adaptive immunity through the generation of indole metabolites and kynurenic acid [32]. These amino acid metabolites, including indole-3-pyruvate (I3P) and 4-hydroxyphenylpyruvate (4HPP), have been shown to suppress ferroptosis induced by agents such as erastin or Ras-selective lethal small molecule 3 (RSL3), significantly reducing lipid peroxidation [11]. The RNA-seq data indicated that I3P and to a lower extent 4HPP induced the expression of a series of mRNAs including those encoding solute carrier family 7, member 11 (SLC7A11), nicotinamide adenine dinucleotide phosphate quinone dehydrogenase 1 (NOQ1), activating transcription factor 4 (ATF4), cytochrome P450 1B1 (CYP1B1), and the Aldo-keto reductase 1C (AKP1C) family, which involved in oxidation irritable reaction and are regulated by the activating transcription factor 4 ATF4, nuclear factor erythroid-derived 2-like 2 (Nrf2), and AHR pathways [11]. In addition to their role in antioxidative gene networks, I3P and 4HPP also exhibit the free radical scavenging properties [11]. In another study, kynurenine—produced by the tryptophan metabolism pathway—has been shown to suppress ferroptotic cell death by scavenging reactive oxygen species (ROS) and activating an Nrf2-dependent, AHR-independent cell-protective pathway, including SLC7A11 to propagate anti-ferroptotic signaling [64]. Cui *et al*. [65] has found that L-kynurenine generated by gastric cancer (GC) cells triggered natural killer (NK) cell ferroptosis in an AHR-independent manner, which suggested that kynurenine plays a dual role depending on the specific circumstances. Collectively, these findings illustrate that IL4I1 can significantly influence cellular homeostasis and control multiple pathways that protect against ferroptotic cell death.

## 6. Potential Role of IL4I1 in Myocardial Infarction

An ischemia injury of the myocardium, MI is primarily caused by the disruption of atherosclerotic plaques, which interrupts coronary flow [66]. The inflammatory response following MI, mediated by diverse immunoreactive cells, is crucial for cardiac repair and remodeling [67]. Recent experimental evidence increasingly supports the essential role of Tregs in the healing processes following MI [68, 69, 70, 71]. Tregs, known for their immune-suppressive properties, are highly enriched in the injured myocardium as early as three days after infarction [69]. These Tregs are initially primed in heart-draining mediastinal lymph nodes, dependent on TCR activation [69, 72], and become fully matured within the myocardial infarction microenvironment, and perform reparative functions [73].

**Fig. 2.**
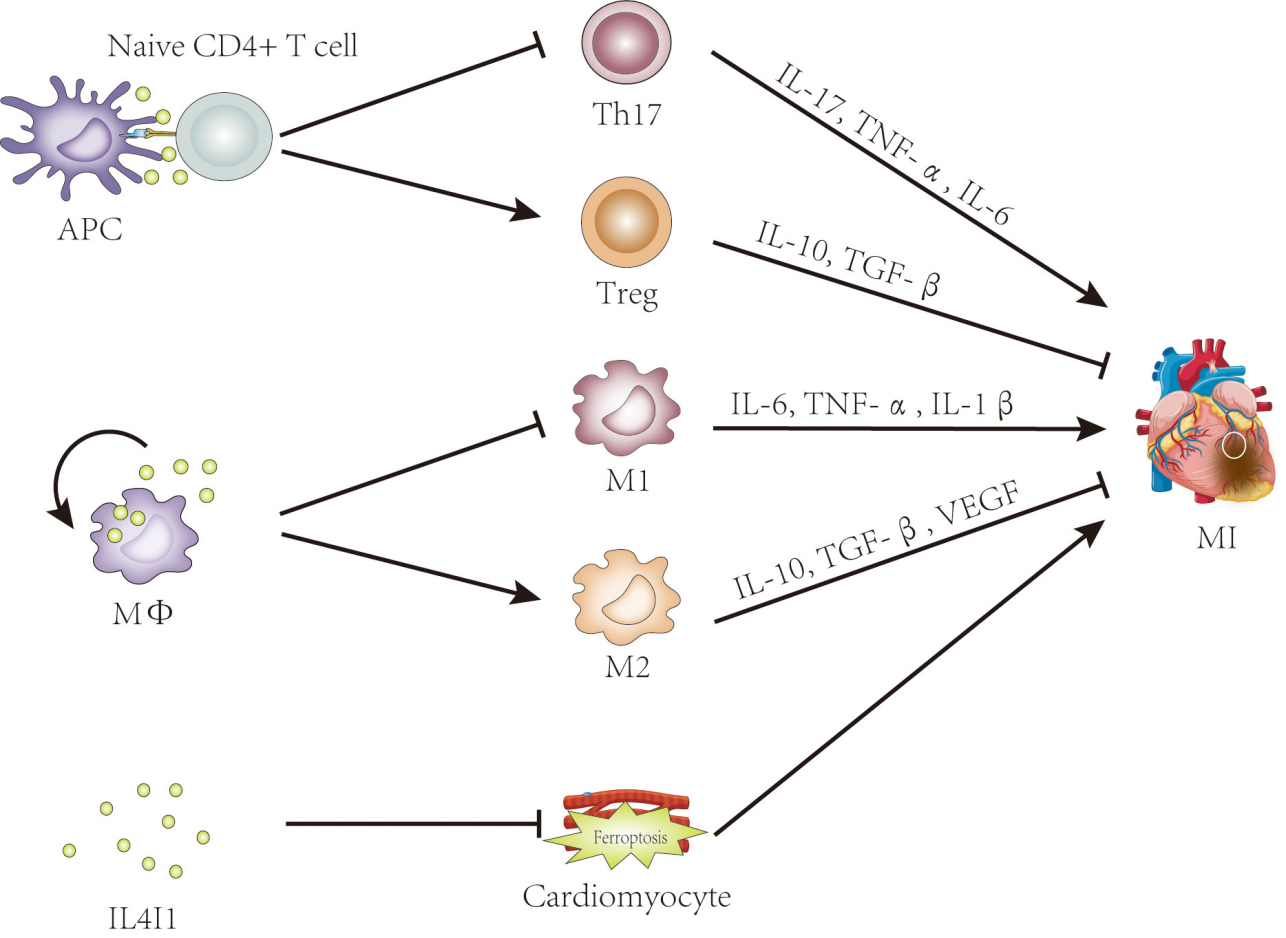
**The potential roles of IL4I1 in myocardial infarction**. Evidence suggests that IL4I1 may influence myocardial infarction by regulating the Th17/Treg balance, promoting M2 macrophage polarization, and inhibiting ferroptosis. IL4I1, IL-4-induced gene 1; APC, antigen-presenting cell; Treg, regulatory T cell; MФ, macrophage; M1, classically activated macrophage; M2, alternatively activated macrophage; MI, myocardial infarction; TNF-α, tumor necrosis factor-α; IL, interleukin; TGF-β, transforming growth factor-β; VEGF, vascular endothelial growth factor; Th17, T helper 17.

In contrast, Th17 cells, though present as minor populations, have received less attention. Clinical research has demonstrated that an imbalance in the Treg/Th17 ratio is involved in the pathogenesis of acute coronary syndrome (ASC), and this ratio has shown high specificity and sensitivity as a predictive indictor for ASC [74, 75, 76]. In short, the Treg/Th17 balance plays a pivotal role in myocardial infarction. The enzyme IL4I1 belonging to the LAAO family, plays an immunosuppressive role in immune cells and immune responses [9, 10, 30]. Previous studies have shown that IL4I1 facilitates Treg differentiation but inhibits Th17 cell differentiation from primary CD4+ T cells [9, 10, 14]. Altogether, these data suggest that IL4I1 is a promising therapeutic target for MI treatment, exerting its effects by regulating Treg/Th17 ratio (Fig. 2).

Macrophages, another key category of cells involved with MI, modulate the balance between pro- and anti-inflammatory responses. Extensive research has demonstrated that both M1 and M2 macrophages are essential in repairing the damaged myocardium after MI through chemical depletion or the knockout of differentiation-related genes [77, 78]. In the early stages (1–3 days post-infarction), M1 macrophages become a major subset of cells in the area, and predominantly focus on removing necrotic cells, while M2 macrophages become the dominant subset after 5 days, aiding in myocardial tissue rebuilding, angiogenesis regulation, and fibrosis promotion [79]. However, continuous activation of M1 macrophages or inhibition of M2 macrophage polarization was found to aggravate myocardial injury and exacerbate cardiac dysfunction following MI [80, 81]. Thus, appropriate regulation of the M1/M2 macrophage phenotypic transformation is essential for cardiac healing [82]. In addition to influencing CD4+ T cells, IL4I1 also effects macrophages by promoting M2 polarization [8]. Given its functions on both T cells and macrophages, IL4I1 may represent a novel target for MI treatment and improving patient prognosis.

Ferroptosis has recently been identified as a significant factor in MI occurrence and progression [83]. Emerging evidence suggests that inhibiting ferroptosis can alleviate the myocardial ischemic injury associated with myocardial infarction [84, 85]. Overall, IL4I1 has been shown to exert anti-ferroptosis effects through multiple pathways, indicating its potential as a therapeutic target for MI.

Although the role of IL4I1 has been well described in various contexts such as pathogenesis of tumor immune escape, defense against bacterial infection, and several models of autoimmune diseases, relatively little known about the role of IL4I1 in the heart following infarction. Yan *et al*. [12] found significantly lower levels of *IL4I1* mRNA expression in peripheral blood mononuclear cells from patients with acute myocardial infarction compared with the stable angina and control groups, suggesting a potential association between IL4I1 and MI. Consistent with these findings, microarray analysis has revealed that IL4I1 is involved in intercellular signaling and interaction, cell movement, and immune cell trafficking in the post-ischemic myocardium [86]. Additionally, adherence to a Mediterranean diet, which has demonstrated favorable effects on cardiovascular risk, has been found to correlate with *IL4I* gene methylation [87]. Moreover, our research group discovered that IL4I1 mediates the suppressive effects of CD4+LAP+ Tregs in the context of atherosclerosis, a common cause of acute myocardial infarction [88]. Therefore, the regulation of the effect of IL4I1 on the immune system and injured myocardium might offer a new direction for limiting MI size, preventing adverse left ventricular remodeling, and improving clinical outcomes following MI. However, the precise role and mechanism of IL4I in MI, whether in animal models or humans, requires further investigation.

## 7. Conclusions

The IL4I1 enzyme performs a critical role in regulating immune responses through diverse mechanisms and has been studied extensively in cancers and inflammatory diseases. Expression levels of IL4I1 are strongly associated with myocardial infarction, suggesting a potential role in disease progression. Overall, further research is needed to explore the functions and molecular mechanisms of IL4I1 in the context of MI.
